# Identification and molecular cloning of glutamate decarboxylase gene from *Lactobacillus casei*

**Published:** 2015-09

**Authors:** Yasaman Tavakoli, Abolghasem Esmaeili, Mohammad Rabbani

**Affiliations:** 1 Department of Biotechnology, Faculty of Advanced Sciences and Technologies, University of Isfahan, Isfahan, Iran; 2 Department of Biology, Faculty of Sciences, University of Isfahan, Isfahan, Iran

**Keywords:** Gamma-aminobutyric acid, Glutamate decarboxylase, *Lactobacillus casei*

## Abstract

Gamma-amino butyric acid (GABA) possesses several physiological functions such as neurotransmission, induction of hypotension, diuretic and tranquilizer effects. Production of GABA-enriched products by lactic acid bacteria has been a focus of different researches in recent years because of their safety and health-promoting specifities. In this study, glutamate decarboxylase (*gad) *gene of a local strains *Lactobacillus casei *was identified and cloned. In order to clone the *gad *gene from this strain, the PCR was carried out using primers designed based on conserved regions. The PCR product was purified and ligated into PGEM-T vector. Comparison of obtained sequences shows that this fragment codes the pyridoxal 5′-phosphate binding region. This strain could possibly be used for the industrial GABA production and also for development of functional fermented foods. *Gad *gene manipulation can also either decrease or increase the activity of enzyme in bacteria.

## INTRODUCTION

Glutamic acid decarboxylase (GAD) produces gamma-aminobutyric acid (GABA), a four carbon amino acid that lacks in protein structure through decarboxylation of glutamate. Activity of this enzyme depends on vitamin B6 and sufficient level of sulfate ions and pyridoxal 5′-phosphate (PLP) cofactor are needed for this process [1]. This enzyme exists in two isoforms with molecular weights of 65 and 67 kilo Dalton in mammals brain [2]. Various microorganisms such as bacteria, fungi and yeasts can produce GABA [3]. Also, GABA is found in extreme quantities among plants, animals and microorganisms as well as human body. GABA with molecular formula of C4H9NO2 and molecular weight of 103.12 g/mole is currently the most important inhibitory neurotransmitter in the central nervous system of mammals [4, 5]. GABA prevents diabetic conditions, and reduces inflammation and blood pressure. GABA also plays various roles in neurotransmission, diuretic, modulation of cardiovascular functions, induction of hypotension and stimulation of immune cells. It is believed that GABA could be used for treatments of Parkinson's disease, stiff-man syndrome, sleeplessness, depression, schizophrenia, cancer and small airway-derived lung adenocarcinoma [6, 7].

GABA is very beneficial in food and pharmaceutical industries; therefore, identifying bacteria that are capable of producing GABA is of great importance [6]. Many varieties of bacteria such as *Bacillus megaterium *[8, 9], *Listeria monocytogenes *[3], *Escherichia coli *[10] and most of lactic acid bacteria (LAB) could produce GABA [7]. Recently, identification of lactic acid bacteria containing *gad *gene and GABA production has become the main interest for investigations in this area. It is because these bacteria are important groups of gram positive bacteria that which are broadly used in food industries as safe products [5, 6]. Lactic acid bacteria with high glutamic acid decarboxylase activity level are also potentially considered as probiotics [11]. Therefore, the first step in identifying these bacteria is to see whether they contain *gad *gene.

The aim of this study was identification and molecular cloning of *gad *gene from *Lactobacillus casei*. Therefore, in this work the existence of *gad *gene in *Lactobacillus casei *was investigated and a fragment containing this gene was successfully cloned in PGEM-T vector. Accordingly, this bacterium could possibly be used for industrial GABA production. To our knowledge, there are no other reports on cloning of *gad *gene in local *Lactobacillus casei*.

## MATERIALS AND METHODS

*Lactobacillus casei *was purchased from Pastor Institute in Tehran and its genomic DNA was used as a pattern for checking the existence of *gad *gene. This bacterium was cultured in liquid MRS medium at 37○C for 24 hours. Genomic DNA was extracted from *Lactobacillus casei *by DNA isolation kit (Qiagen, Inc, CA), according to the manufacturers protocol. Extracted DNA quality was evaluated by means of electrophoresis on 1% agarose gel.

The polymerase chain reaction (PCR) was carried out in a 25 μl volume containing 1.5 μl of genomic DNA as a template, 1 μl of each primer and 5 μl of a PCR master mix (1.25 µl MgCl2, 1 µl dNTPs, 2.5 µl PCR buffer, 0.25 µl Taq DNA polymerase). Sense and antisense oligonucleotide primers of lactic acid bacteria *gad *gene were designed using oligo 7 software based on DNA sequences of above mentioned bacteria recorded in GenBank (Accession No KP178671 and DQ168031). Forward and reversed primers were 5’-ATG GAA AAC ACA CGC ATG AAA C-3’ and 5’- TTA GTG CGT GAA CCC GTA TT-3’ respectively. PCR cycles included a denaturation step at 94○C for 10 min followed by 30 cycles of denaturation at 95○C for 60 sec, annealing of primers at 58○C for 60 sec, extension the desired piece at 72○C for 2 min and final extension stage at 72○C for 10 min. Finally, the PCR products were analyzed by electrophoresis on a 1% agarose gel. Then, the desired band was excised from the gel and purified using a DNA Extraction Kit (Fermentas) according to manufacturer instructions

In order to clone *gad *gene in pGEM-T plasmid (Promega, Madison, WI), PCR product and the plasmid were mixed in a sterile micro-tube with a ratio of 3 to 1, respectively. Then, 5 µl of ligation buffer plus 1 µl of ligase enzyme was added to the micro-tube. Then the mixture was kept at room temperature for 1 hour and then 24 hours at 4○C. *E.coli *XL1 Blue (Novagen, Inc., San Diego, CA, USA) was used as host for cloning and recombinant plasmid reproduction. Calcium chloride method was used to turn these bacteria to competent cells [12] and then the DNA was transformed to these cells through heat-shock method [13]. After transformation, these bacteria were grown onto LB agar plates containing 100 μg of ampicillin per ml and 1 mM IPTG and X-Gal followed by incubation at 37○C for 24 hour. Then, from each agar plate several white colonies were randomly selected and inoculated in a LB medium having ampicillin and incubated at 37○C for 24 hour. Using a plasmid extraction kit (Fermentas, Iran, Tehran), and following its manual, recombinant plasmids (pGEMT- GAD) were extracted from white colonies and PCR was performed (same procedure as mentioned before) for verification. The pGEMT-GAD plasmids were then sent to Faza Biotech Company (Iran, Tehran) for sequencing and to obtain more accuracy, the process was performed in two directions using SP6 Reverse and T7 Forward primers. The results were then compared to those existing fragments in the NCBI server (http://ncbi.nlm.nih.gov/ BLAST) using BLAST software.

## RESULTS AND DISCUSSION

Based on agarose gel analysis, DNA fragments had acceptable quality and yield. PCR reaction for *gad *gene amplification was designed with exclusive primers in a manner that the full length of gene could be reproduced. The products of PCR reaction were checked through electrophoresis on 1% agarose gel and as it was expected, a piece with a length of approximately 1398 base pair (bp) was observed which matches the *gad *gene of other lactic acid bacteria in terms of length. The PCR products were ligated into a pGEMT standard cloning vector. The plasmid (pGEMT-GAD) was transformed successfully into *E.coli*. The validity of cloning process was verified by performing electrophoresis on 1% agarose gel and formation of a 1398 bp piece. The recombinant plasmids from the white colony were sequenced and compared with available sequences in the GenBank. Our data indicated that the length of *gad *gene in *Lactobacillus casei *was 1398 bp.

Lactic acid bacteria (LAB) are safe organisms and usually have the ability to secrete recombinant proteins directly into the culture medium. These bacteria could be suitable candidates for the expression of recombinant proteins [14]. They could also produce high level of GABA. So, these bacteria can be used as safe bioreactors in medicinal and industrial biotechnology.

Identification and molecular cloning of *gad *gene in lactic acid bacteria is of great importance due to the ability of this enzyme in producing GABA which is used in food and pharmaceutical industries [6]. In 1961, for the first time, GAD enzyme was demonstrated to be present in *Escherichia coli *and nearly 30 years later the sequence of this enzyme in *E. Coli *was reported in the gene bank with the accession number of M84025 [10]. Because *Escherichia coli *contains shiga toxin and also it is a pathogen, this bacterium is not a safe candidate for GABA production. Thus, in recent times most investigators consider lactic acid bacteria for producing GABA [15]. Glutamate decarboxylase enzyme of LAB is an intracellular enzyme [16] which contains the same subunits with molecular mass fluctuating from 54 to 62 kDa. However, variation in N- and C- terminal regions of initial structure of GAD enzyme has a significant impact on its capability in GABA production in different types of LAB. For this reason some strains have higher ability to produce GABA [17], while others have not.

The full length of *gad *gene in *Lactobacillus plantarum *KCTC3015 [18], *Lactobacillus paracasei *NFRI7415 [17], *Lactobacillus brevis *IFO12005 [1], *Lactococcus lactis *01-7 [19], *Lactobacillus brevis *BH2 [8], *Lactobacillus brevis *OPK3 [20], and *Streptococcus thermophilus *Y2 [21] has been determined. In this study we cloned the gene encoding GAD of *Lactobacillus casei*. BLAST results showed that *gad *gene from this strain has 98% similarity with *Lactobacillus paracasei *JCM 8130. To find different GABA-producing strains from lactic acid bacteria, especially high- yielding strains, more studies have to be done in future.

## Notes

**Acknowledgements: **The present work is part of MSc thesis that was supported by the grant from University of Isfahan (NO. 97886/90).
